# A Nutritional Counseling Program Prevents an Increase in Workers' Dietary Intake and Body Weight During the COVID-19 Pandemic

**DOI:** 10.3389/fphys.2021.703862

**Published:** 2021-07-21

**Authors:** Patricia A. Nehme, Luisa P. Marot, Luciana F. R. Nogueira, Elaine C. Marqueze, Cibele A. Crispim, Claudia R. C. Moreno

**Affiliations:** ^1^Department of Health, Life Cycles and Society, School of Public Health, University of São Paulo, São Paulo, Brazil; ^2^Federal University of Uberlândia, Uberlândia, Brazil; ^3^Public Health Graduate Program, Catholic University of Santos, Santos, Brazil; ^4^Psychology Department, Stockholm University, Stockholm, Sweden

**Keywords:** dietary intake, nutritional counseling, COVID-19 pandemic, shift work, obesity

## Abstract

The COVID-19 pandemic caused thousands of deaths and changed lives all over the world. Daily life has also altered people's eating habits, mainly among those who stayed working at home. However, changes in the eating habits of workers who remained working during the pandemic are still unknown. The aim of this study was to evaluate the impact of the COVID-19 pandemic on the dietary habits of day and shift workers from a condominium management company, as well as to measure adherence to a nutritional counseling program and its effect on workers' food intake and body weight. One hundred and fifty-one workers (77.5% of day workers and 22.5% of shift workers) were followed up in the pre-pandemic period and during the pandemic. Data on anthropometry, food consumption, and adherence to nutritional counseling were collected during nutritional meetings, which focused on qualitative modification of food intake and control of energy consumption. The rate of adherence to the program did not differ between shifts. The pandemic significantly increased the intake of calories, macronutrients, and several micronutrients in workers of both shifts. Adherence to the nutritional counseling program had an impact on the consumption of proteins and some micronutrients, and also promoted a reduction in body weight and body mass index of workers of both shifts. Evening/night shift workers overall ate their meals later than day workers and also presented an earlier afternoon snack during the pandemic when compared with the pre-pandemic period. In conclusion, the pandemic seems to contribute to the increase in food intake of workers, regardless of the work shift. Those who joined a nutritional counseling program managed their food intake and lost weight.

## Introduction

The first imported COVID-19 case was initially reported in Brazil on February 26, 2020 (Ministério da Saúde, Brasil, 2020). Some weeks later, the WHO decreed the existence of a global pandemic (World Health Organization, 2020). Since then, social distancing has been highly recommended as a preventive measure for disease expansion (Zvolensky et al., 2020). Recent evidence has pointed out to important consequences for health resulting from this measure, with significant psychological impacts (Brooks et al., 2020), implying changes in lifestyle. In this context, the increase in caloric intake and the consumption of a low-quality diet performed in the home environment (Pereira et al., 2020), associated with the decrease in the practice of physical activities, have become common practices (Katsoulis et al., 2021). The sum of the aforementioned factors has become a risk factor for the increase in or worsening of chronic non-communicable diseases such as obesity during the pandemic (Branley-Bell and Talbot, 2020). Unfortunately, growing numbers of reports have connected obesity to more severe COVID-19 illness and death, by mechanisms such as mechanical changes of the airways and lung parenchyma, systemic and airway inflammation, and general metabolic dysfunction that adversely affect pulmonary function and/or response to treatment (Abiri et al., 2021). As the virus continues to spread worldwide, health care professionals should carefully monitor and manage obese patients for prompt and targeted treatment, as well as acting to prevent weight gain among the population.

The changes in eating behavior resulting from social distancing seem to be especially caused by changes in consumption behavior, with an increase in the frequency of online shopping and consumption of fast foods by delivery purchases (Arraes, 2020). Another change in this sense is the search from processed and ultra-processed foods that have greater durability, to the detriment of fresh foods, which have shown a decrease in consumption in recent times (Ammar et al., 2020). In addition, changes in the way of managing day-to-day eating can lead to eating disorders capable of exceeding the individual's daily caloric needs (Hill et al., 2012). Such changes in eating behavior also seem to have occurred in people who continued to work in person, since the domestic eating routine has changed and many restaurants have remained closed, preventing workers from having their meals in these establishments during the workday. This impact was particularly relevant among night and shift workers.

Shift work is defined as any work schedule that does not occur within the usual patterns (Vedaa et al., 2016) and as the main characteristic that has a continuous 24-h operation (Waage et al., 2012). The current “24-h society” usually demands these irregular or atypical working hours, which means working outside of the habitual daytime hours—between 19:00 and 6:00 h (Akerstedt, 1998). Previous studies have shown that shift work is associated with several metabolic and nutritional diseases (Gan et al., 2015; Itani et al., 2017), including obesity (Antunes et al., 2010). Among the nutritional factors that lead to weight gain in shift workers are a poor-quality diet (Balieiro et al., 2014; Bonnell et al., 2017), frequent irregular meals (Bonham et al., 2016; Souza et al., 2019), and, maybe, usual food intake at night (Esquirol et al., 2009).

Nutritional counseling programs have been shown to be scarce among shift workers (Phoi and Keogh, 2019). A literature review conducted by Phoi and Keogh (2019) focused on studying dietary interventions exclusively among night shift workers, and clear conclusions were not possible due to the small number of studies and varied study protocols. Faced with the vulnerability of nutritional health of shift workers compared with day workers (Antunes et al., 2010; Gan et al., 2015; Itani et al., 2017), intervention programs capable of improving the eating habits of individuals and preventing weight gain during the COVID-19 pandemic are considered very important. In view of the aforesaid, this study evaluated the impact of the COVID-19 pandemic on the dietary habits of day and shift workers from a condominium management company, as well as measuring adherence to a nutritional counseling program and its effect on workers' food intake and body weight. Our hypothesis is that the pandemic led to an increase in the food intake of workers in both shifts, but that shift workers would have less adherence to a nutritional counseling program, less positive changes in nutrient intake, and less loss in body weight in comparison with day workers.

## Materials and Methods

### Design and Study Location

This is a quasi-experimental study, a design that intended to estimate the effect of an intervention in the absence of randomization (Harris et al., 2006). The study was approved by the Research Ethics Committee of the Faculty of Public Health of the University of São Paulo (Process# 4298715). Workers were informed of the purpose of the study and consented to the use of the data. Data were collected between November 2019 and February 2020 (period pre-pandemic) and between July and August 2020 (during the pandemic). The inclusion criteria in the study were being a duly registered worker in the company and having attended a nutrition consultation in the period before and during the pandemic.

The study was carried out in a condominium management company in a city on the northern coast of the state of São Paulo, Brazil. The condominium is in a subdivision of ~9 million m^2^ and has 10,000 houses. More than 2,000 people are permanent residents and about 25,000 constitute the floating population (summer).

### Study Sample

The condominium management company had 527 employees at the time of the study, among which 464 were men (88%). Data from 151 individuals (108 males; 28.65% of the company's population) who voluntarily participated in a nutritional counseling program offered by the company were analyzed. Among the workers, 77.5% (*n* = 117) performed their activities in the day shift (anytime between 06:00 and 17:00 h), 9.3% (*n* = 14) in the evening (anytime between 14:00 and 23:59 h), and 13.2% (*n* = 20) during the night (anytime between 00:00 and 08:00 h). Demographic information of the workers (age, sex, occupation/position, work hours) and anthropometric data (weight and height) available in the workers' register or collected during the nutritional consultation were included in the study. The power of the sample was calculated a *posteriori* using G^*^Power software, with the repeated measures test (pre and pandemic) as a reference, the total number of participants (150), four groups (day and evening/night, adhered to the program and did not adhere the program), and an effect size of 0.25. The analysis indicated that the studied sample was sufficient with a statistical power sample of 0.99.

### Nutritional Counseling Program Protocol

Nutritional counseling followed a standard individual care protocol, which aimed to reduce the rate of withdrawal from the treatment. A strategy is developed in which the workers became autonomous, able to identify the difficulties to exercise self-control over food intake, as well as individual mechanisms to overcome them. There is no need to present diseases since the company's views are to offer this service to promote health. The data used in our study followed the company's regular procedure with the appointment made in the previous week by the social assistant. On the day of the appointment, the workers have measured their anthropometric measurements and have been evaluated by the nutritionist for around 1 h (including clinical history and presence of comorbidities). Pre- and pandemic consultations took place 3 months apart. The same nutritionist attended to the workers on both occasions.

The qualitative characteristics of the dietary pattern in the first and second appointments were evaluated by a 24 h food recall in order to verify the consumption of fruits, vegetables, and ultra-processed foods, through open questions to better understand the food phenomenon. Questions related to the role of food in health were also used as a way of disseminating knowledge, as well as motivating the participant regarding treatment adherence.

In addition, during the appointment, basic concepts of food groups, principles of healthy eating, how to prepare a healthy dish, and fashionable diets were addressed, in addition to the importance of physical activity. Qualitative modification of food intake and control of energy consumption and more appropriate times for meals throughout the day were also addressed. An individualized food plan was developed with the worker's participation with regard to food preferences. The food plan was qualitative/quantitative according to the individual estimates of energy expenditure and also according to the pathology presented, when applicable.

### Data Collection

The 24 h recall (24 hR) was used in order to assess the food consumption. This method describes a wide variety of foods, which is easy and quick to make and provides no change in eating behavior. In addition, the method minimizes the error in calculating the prevalence of nutrient inadequacy, because it considers the random characteristics of the diet as a cutoff point (Slater et al., 2004). The 24 hR assessed detailed information about all foods and beverages that were consumed in the past 24 h prior to their application. A visual and photographic atlas was used as a visual resource to estimate the portions and quantities consumed. A single 24 hR was collected prior to the pandemic period (between 1 and 4 months before the pandemic) and another one during the pandemic period. According to Thompson and Subar (2013), a single 24 hR can be used to describe the average food intake of a population. In order to monitor the quality of the interviews and standardize quantities and recipes, the data were checked individually regarding quantity, form of preparation, trademarks, and type of preparation, prior to the organization of the database.

Weight was measured on a digital scale with an accuracy of 0.1 kg with a capacity of 150 kg, properly calibrated at regular intervals of use (World Health Organization, 2000). Height was measured using a fixed stadiometer with an accuracy of 0.1 cm. Body mass index (BMI, kg/m^2^) was calculated as the weight (kg) divided by the height squared (m^2^).

Energy and nutrient intake were analyzed by the Virtual Nutri program (Philippi et al., 1996). Additionally, we sought to analyze the meal timing. Amount and distribution of energy intake as well as average total food consumption (in total grams and total kilocalories), and macro- and micronutrient intake (total protein, fat, carbohydrates fibers, minerals, and vitamins) were calculated pre- and during the pandemic period for every single meal.

### Statistical Analysis

Statistical analyses were performed using the software STATA 12.0 and Statistica 7. Significance level was set at 5%. The study sample will be presented in day workers (77.5%; *n* = 117) and shift workers, in which evening and night workers (22.5%; *n* = 34) were grouped.

Pearson's chi-squared test was performed to evaluate the proportion of sociodemographic and health variables by work shift and adherence to the nutrition program. In order to compare the averages of anthropometric variables by work shift and adherence to the nutrition program, the Mann–Whitney test was performed.

General linear model (GLM) was performed in order to evaluate the effect of the interaction between pandemic period (pre-pandemic and during pandemic) and shift condition (day shift and evening/night shift) on meal timing (dependent variable), with adjustments for age and sex. GLM was also performed to evaluate the meal timing in the pre-pandemic period and during the pandemic per work shift, adjusted for age and sex. *Post hoc* pairwise comparisons were performed using LSD. In all tests, *p* < 0.05 was considered statistically significant.

## Results

Evening and night workers were younger (34.5 y ± 9.7 y) than day workers (42.8 y ± 10.2 y). There were no differences in sociodemographic and health variables neither according to the shift nor in relation to who adhered or not to the nutrition program (Table 1).

**Table 1:** Sociodemographic variables by work shift and adherence to the dietary program.

**Sociodemographic and health variables**	**Day worker**	**Evening/night worker**	**Pearson's chi-square**	**Adhered the program**	**Did not adhere the program**	**Pearson's chi-square**
	***n(%)***	***n(%)***	***p-value***	***n(%)***	***n(%)***	***p-value***
**Sex**						
Female	80 (69.0)	27 (79.4)		33 (76.7)	75 (69.4)	
Male	36 (31.0)	7 (20.6)	0.24	10 (23.3)	33 (30.6)	0.37
**Work sector**						
Administrative	50 (43.1)	0 (0)		17 (39.5)	33 (30.6)	
Inspection/Security	21 (18.1)	31 (91.2)		10 (23.3)	43 (39.8)	
Sanitation/Maintenance	45 (38.8)	3 (8.8)	…	16 (37.2)	32 (29.6)	0.16
**Shift**						
Day				34 (79.1)	82 (76.6)	
Evening/night			…	9 (20.9)	25 (23.4)	0.75
**Reason to get in the nutrition program**						
Weight loss/healthy diet/sports practice	100 (86.2)	34 (100)		36 (83.7)	99 (91.7)	
Disease/altered biochemical exams	16 (13.8)	0 (0)	…	7 (16.3)	9 (8.3)	0.15
**Adherence in the program**						
Yes	34 (29.3)	9 (26.5)				
No	82 (70.7)	25 (73.5)	0.75			…
**Reason for adhering the program**						
Easy meal plan/motivation	33 (28.5)	8 (23.5)		41 (95.4)	0 (0)	
Pandemic	41 (35.3)	10 (29.4)		1 (2.3)	51 (47.2)	
Anxiety/difficulty planning/lack of motivation	42 (36.2)	16 (47.1)	0.52	1 (2.3)	57 (52.8)	…

There were no statistically significant differences in body weight and BMI according to the shift in any of the periods analyzed (before or during the pandemic). There was also no difference in the delta of body weight or in the BMI before and after the pandemic. On the other hand, when evaluating these variables among the workers who adhered to the nutrition program, those who adhered had a greater reduction in body weight and BMI (Table 2).

**Table 2:** Description of anthropometric variables by work shift and adherence to the dietary program.

**Anthropometric variables**	**Day workers**	**Evening/night workers**	**Mann–Whitney**	**Adhered the program**		**Mann–Whitney**
	**Mean (SD)**	**Mean (SD)**	***p-value***			***p-value***
				**Yes**	**No**	
Weight (kg) pre-pandemic	80.5 (15.4)	82.5 (13.6)	0.40			…
Weight (kg) pandemic	81.5 (15.7)	83.4 (14.1)	0.46	72.3 (11.9)	85.8 (14.8)	** < 0.01**
BMI (kg/m^2^) pre-pandemic	27.9 (4.2)	27.8 (3.8)	0.88			…
BMI (kg/m^2^) pandemic	28.3 (4.3)	28.0 (4.0)	0.77	25.2 (2.7)	29.4 (4.1)	** < 0.01**

*Bold values mean statistically significant*.

Analysis of the interaction between shift vs. pandemic showed that day workers increased the consumption of polyunsaturated fats from pre-pandemic to during pandemic period, while night workers did not change the intake of this nutrient (LSD *p* < 0.01) (Table 3). The other results (Table 3) show the isolated effect of the pandemic on increasing energy intake, macronutrients, saturated, monounsaturated and polyunsaturated fat, cholesterol, as well as for several vitamins (A, B2, B5, pantothenic acid, B12, C, E, niacin) and minerals (copper, folate, phosphorus, magnesium, potassium, sodium, zinc). A significant isolated effect of the shift was observed for the consumption of vitamins B12, C, E, and folate (more details in Supplementary Table 1).

**Table 3:** Generalized linear model of the variables related to the consumption of nutrients in the pre-pandemic period and during the pandemic per work shift, adjusted for age and sex.

**Nutrients**	**Pre-pandemic**				**Pandemic**				**Shift effect**	**Pandemic effect**	**Interaction Shift x Pandemic**
	**Day**		**Evening/night**		**Day**		**Evening/night**				
	**Mean**	**SE**	**Mean**	**SE**	**Mean**	**SE**	**Mean**	**EP**			
Energy (kcal)	1,261.5	57.3	1,406.5	105.3	1,799.6	57.3	1,886.8	105.3	*p =* 0.79	***p<*** **0.01**	*p =* 0.73
Protein (g)	72.6	4.4	84.1	8.0	103.2	4.4	116.0	8.0	*p =* 0.48	***p<*** **0.01**	*p =* 0.92
Carbohydrate (g)	152.5	7.1	152.7	13.0	200.5	7.1	200.9	13.0	*p =* 0.37	***p<*** **0.01**	*p =* 1.00
Total fiber (g)	13.1	0.7	12.7	1.3	16.3	0.7	14.9	1.3	*p =* 0.46	***p=*** **0.01**	*p =* 0.63
Insoluble fiber (g)	2.0	0.2	1.7	0.3	2.2	0.2	1.9	0.3	*p =* 0.50	*p =* 0.52	*p =* 0.99
Soluble fiber (g)	1.2	0.1	1.1	0.2	1.4	0.1	1.1	0.2	*p =* 0.49	*p =* 0.54	*p =* 0.72
Total fat (g)	40.1	2.7	52.7	4.9	63.9	2.7	64.5	4.9	*p =* 0.80	***p<*** **0.01**	*p =* 0.13
Saturated fat (g)	17.0	1.3	21.6	2.4	25.0	1.3	27.1	2.4	*p =* 0.70	***p<*** **0.01**	*p =* 0.52
Monounsaturated fat (g)	13.8	1.0	18.2	1.9	20.5	1.0	21.4	1.9	*p =* 0.93	***p<*** **0.01**	*p =* 0.24
Polyunsaturated fat (g)	7.6	0.7	11.8	1.3	12.6	0.7	12.1	1.3	*p =* 0.77	***p=*** **0.01**	***p=*** **0.03**
Cholesterol (mg)	269.8	27.1	377.1	49.8	415.9	27.1	480.7	49.8	*p =* 0.44	***p<*** **0.01**	*p =* 0.60
Vitamin A (UI)	370.8	104.9	302.2	192.9	798.1	104.9	498.0	192.9	*p =* 0.10	***p=*** **0.04**	*p =* 0.46
Vitamin B1 (mg)	1.0	0.1	1.1	0.2	1.4	0.1	1.3	0.2	*p =* 0.87	*p =* 0.08	*p =* 0.56
Vitamin B2 (mg)	0.7	0.0	0.8	0.1	1.0	0.0	1.1	0.1	*p =* 0.18	***p<*** **0.01**	*p =* 0.90
Pantothenic acid (mg)	1.0	0.1	1.0	0.1	1.2	0.1	1.6	0.1	*p =* 0.21	***p<*** **0.01**	*p =* 0.10
Vitamin B6 (mg)	0.7	0.1	0.6	0.1	0.9	0.1	1.2	0.1	*p =* 0.33	***p<*** **0.01**	*p =* 0.06
Vitamin B12 (mg)	1.2	0.3	1.8	0.5	1.6	0.3	2.8	0.5	***p=*** **0.04**	*p =* 0.07	*p =* 0.46
Vitamin C (mg)	54.6	20.0	132.7	36.8	64.0	20.0	199.6	36.8	***p<*** **0.01**	*p =* 0.22	*p =* 0.33
Vitamin D (μg)	1.2	0.9	1.5	1.6	2.7	0.9	3.9	1.6	*p =* 0.77	*p =* 0.13	*p =* 0.75
Vitamin E (mg)	3.9	0.5	3.2	1.0	6.1	0.5	4.1	1.0	***p=*** **0.01**	***p=*** **0.05**	*p =* 0.38
Niacin (mg)	15.3	1.4	13.4	2.5	22.1	1.4	22.6	2.5	*p =* 0.54	***p<*** **0.01**	*p =* 0.55
Calcium (mg)	392.1	30.1	458.3	55.4	493.3	30.1	512.4	55.4	*p =* 0.40	*p =* 0.08	*p =* 0.60
Copper (mg)	0.7	0.1	0.8	0.1	1.0	0.1	1.0	0.1	*p =* 0.17	***p=*** **0.02**	*p =* 0.66
Folate (μg)	47.3	4.9	61.5	9.0	58.2	4.9	95.8	9.0	***p<*** **0.01**	***p<*** **0.01**	*p =* 0.11
Iron (mg)	52.4	15.6	77.0	28.6	26.2	15.6	12.6	28.6	*p =* 0.77	***p=*** **0.05**	*p =* 0.41
Phosphor (mg)	855.5	51.0	990.5	93.9	1,198.4	51.0	1,274.5	93.9	*p =* 0.58	***p<*** **0.01**	*p =* 0.70
Iodine (μg)	5.3	1.2	2.7	2.3	6.1	1.2	2.4	2.3	*p =* 0.16	*p =* 0.89	*p =* 0.76
Magnesium (mg)	159.3	7.0	164.6	12.9	201.9	7.0	213.2	12.9	*p =* 0.67	***p<*** **0.01**	*p =* 0.77
Manganese (mg)	1.5	0.2	1.1	0.3	1.8	0.2	1.3	0.3	*p =* 0.11	*p =* 0.29	*p =* 0.64
Potassium (mg)	1,745.6	77.8	1,812.9	143.0	2,241.5	77.8	2,445.8	143.0	*p =* 0.33	***p<*** **0.01**	*p =* 0.55
Selenium (μg)	28.9	2.9	32.5	5.4	36.0	2.9	31.1	5.4	*p =* 0.89	*p =* 0.52	*p =* 0.33
Sodium (mg)	1,770.2	122.0	2,206.7	224.3	2737.5	122.0	2,556.9	224.3	*p =* 0.83	***p<*** **0.01**	*p =* 0.09
Zinc (mg)	9.0	0.8	10.8	1.5	12.2	0.8	15.4	1.5	*p =* 0.36	***p<*** **0.01**	*p =* 0.56

*More details of isolated effects of the pandemic and work shift in Supplementary Table 1. Bold values mean statistically significant*.

**Figure 1:**
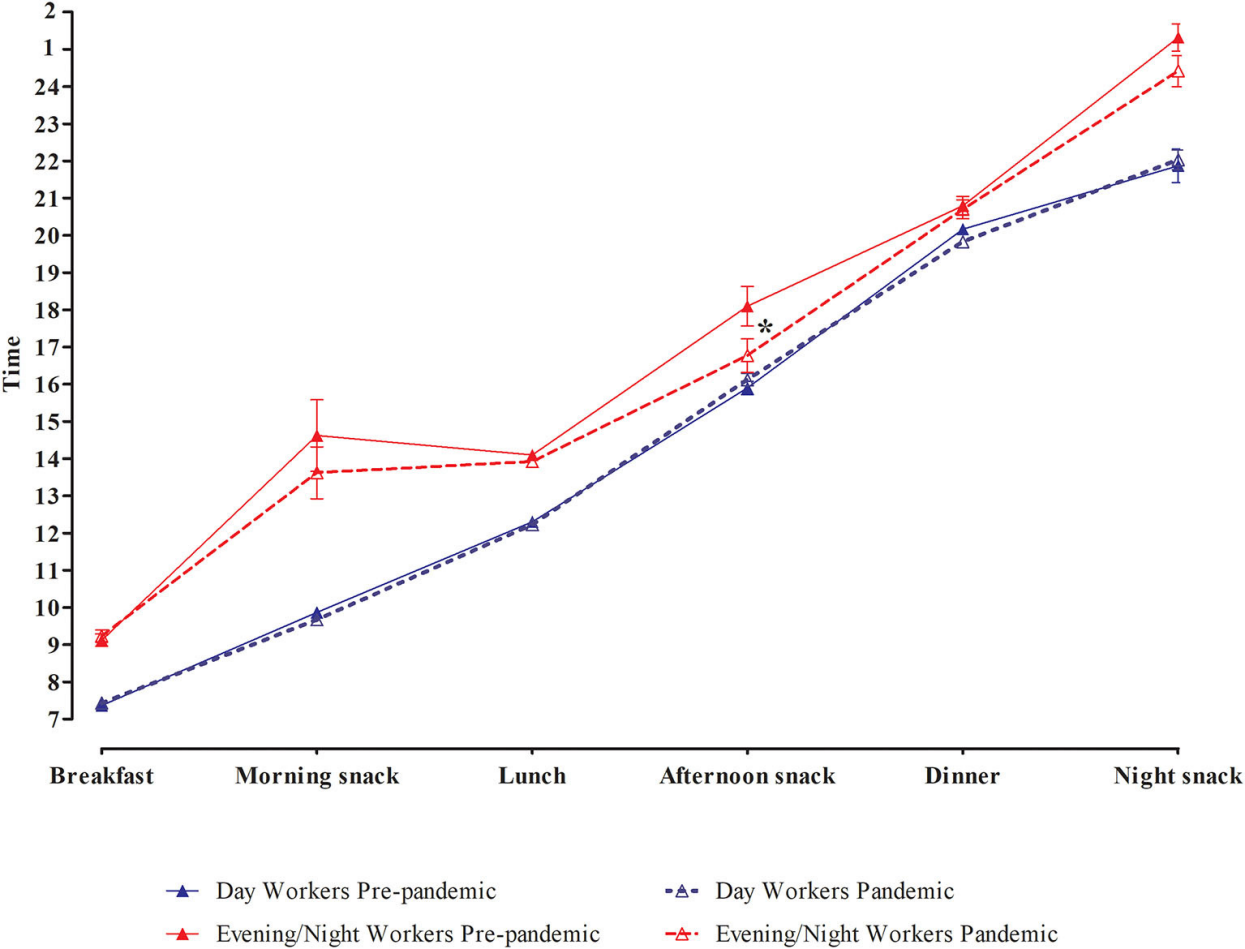
Generalized linear model of meal timing in the pre-pandemic period and during the pandemic per work shift, adjusted for age and sex. Values are present as mean ± SE. More details in Supplementary Table 5.

**Figure 2:**
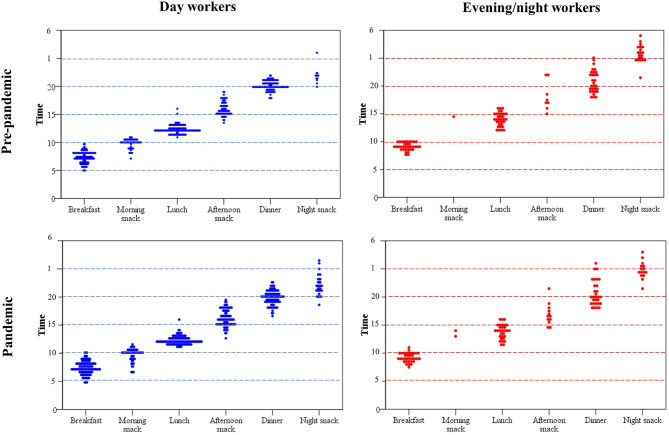
Dispersion of meal timing in the pre-pandemic period and during the pandemic per work shift.

The GLM test showed an interaction effect between shift and pandemic period on meal timing, as well as an isolated effect of shift (Figure 1). The isolated effect of the shift showed that, in general, evening/night shift workers ate their meals 2 h later than day workers (*p* < 0.01) (see Supplementary Table 5). In addition, the effect of the interaction between shift and pandemic period showed that evening/night workers ate the afternoon snack earlier during pandemic (16:46 ± 0:27) than pre-pandemic period (18:07 ± 0:32; *p* = 0.03), while day workers maintained their usual meal timings (16:07 ± 0:10 and 15:54 ± 0:11, respectively; *p* > 0.05) (see Supplementary Table 5). Figure 2 shows the dispersion of meal timing in the pre-pandemic period and during the pandemic per work shift.

Table 4 shows the variables related to the consumption of nutrients in the pandemic period according to shift, adherence to nutritional counseling, and their interaction. The results show an isolated effect of shift on the intake of vitamins B6, B12, C, and folate, with higher values of all these nutrients among shift workers compared with day workers. In addition, an isolated effect of adherence to the program was found in the intake of vitamins B6, B12, C, magnesium, potassium, and zinc, with higher values of consumption of these nutrients among those who adhered to the program (Table 4; for more details, see Supplementary Table 2).

**Table 4:** Generalized linear model of variables related to nutrient consumption during the pandemic period according to adherence to dietary program per work shift, adjusted for age and sex.

**Eating variables**	**Adhered the program**				**Did not adhere the program**				**Shift effect**	**Effect of adherence to the program**	**Interaction shift × adherence**
	**Day**		**Evening/night**		**Day**		**Evening/night**				
	**Mean**	**SE**	**Mean**	**SE**	**Mean**	**SE**	**Mean**	**SE**			
Energy (kcal)	1,722.5	125.0	2,150.5	243.0	1,832.0	81.0	1,791.8	145.8	*p =* 0.88	*p =* 0.37	*p =* 0.20
Protein (g)	109.8	9.0	142.1	17.4	100.4	5.8	106.6	10.4	*p =* 0.68	***p=*** **0.05**	*p =* 0.33
Carbohydrate (g)	186.9	15.1	202.4	29.3	206.2	9.8	200.3	17.6	*p =* 0.68	*p =* 0.64	*p =* 0.64
Total fiber (g)	17.4	1.6	16.0	3.0	15.9	1.0	14.5	1.8	*p =* 0.65	*p =* 0.53	*p =* 1.00
Insoluble fiber (g)	2.3	0.3	1.7	0.7	2.1	0.2	2.0	0.4	*p =* 0.66	*p =* 0.76	*p =* 0.68
Soluble fiber (g)	1.4	0.2	1.0	0.4	1.3	0.1	1.2	0.3	*p =* 0.53	*p =* 0.77	*p =* 0.61
Total fat (g)	58.9	6.1	70.9	11.8	66.0	3.9	62.1	7.1	*p =* 0.39	*p =* 0.69	*p =* 0.38
Saturated fat (g)	23.0	2.9	24.5	5.6	25.8	1.9	28.1	3.3	*p =* 0.58	*p =* 0.50	*p =* 0.83
Monounsaturated fat (g)	19.1	2.2	23.3	4.3	21.1	1.4	20.7	2.6	*p =* 0.49	*p =* 0.80	*p =* 0.52
Polyunsaturated fat (g)	11.7	1.6	13.4	3.2	13.0	1.1	11.7	1.9	*p =* 0.56	*p =* 0.74	*p =* 0.49
Cholesterol (mg)	399.3	56.1	451.4	109.0	422.9	36.3	491.3	65.4	*p =* 0.93	*p =* 0.72	*p =* 0.82
Vitamin A (UI)	668.1	265.0	363.6	515.0	852.7	171.7	546.4	309.0	*p =* 0.21	*p =* 0.68	*p =* 0.97
Vitamin B1 (mg)	1.6	0.2	1.5	0.3	1.2	0.1	1.2	0.2	*p =* 0.83	*p =* 0.15	*p =* 0.81
Vitamin B2 (mg)	0.9	0.1	1.4	0.2	1.0	0.1	1.0	0.1	*p =* 0.16	*p =* 0.13	*p =* 0.06
Vitamin B6 (mg)	1.0	0.1	1.8	0.2	0.8	0.1	0.9	0.1	***p=*** **0.02**	***p<*** **0.01**	***p=*** **0.03**
Vitamin B12 (mg)	1.8	0.5	6.1	1.0	1.6	0.3	1.6	0.6	***p<*** **0.01**	***p*** **<** **0.01**	***p<*** **0.01**
Vitamin C (mg)	71.2	36.8	370.5	71.5	61.0	23.8	138.0	42.9	***p<*** **0.01**	***p=*** **0.02**	***p=*** **0.02**
Vitamin D (μg)	1.9	2.3	1.0	4.4	3.0	1.5	4.9	2.7	*p =* 0.91	*p =* 0.38	*p =* 0.61
Vitamin E (mg)	5.7	1.2	3.8	2.3	6.3	0.8	4.2	1.4	*p =* 0.10	*p =* 0.80	*p =* 0.97
Niacin (mg)	22.5	2.9	28.7	5.6	21.9	1.9	20.4	3.4	*p =* 0.96	*p =* 0.20	*p =* 0.34
Calcium (mg)	462.2	68.3	484.8	132.8	506.4	44.3	522.3	79.7	*p =* 0.90	*p =* 0.68	*p =* 0.97
Copper (mg)	0.9	0.2	1.2	0.4	1.1	0.1	0.9	0.2	*p =* 0.46	*p =* 0.67	*p =* 0.28
Pantothenic acid (mg)	1.4	0.2	2.1	0.3	1.1	0.1	1.4	0.2	*p =* 0.05	*p =* 0.06	*p =* 0.36
Folate (μg)	64.9	10.2	114.8	19.8	55.4	6.6	88.9	11.9	***p<*** **0.01**	*p =* 0.24	*p =* 0.52
Iron (mg)	45.2	18.5	16.0	36.0	18.1	12.0	11.3	21.6	*p =* 0.40	*p =* 0.42	*p =* 0.64
Phosphor (mg)	1,301.0	102.5	1,469.9	199.1	1,155.4	66.4	1,204.2	119.5	*p =* 0.96	*p =* 0.13	*p =* 0.73
Iodine (μg)	10.1	2.4	2.2	4.7	4.4	1.6	2.4	2.8	*p =* 0.20	*p =* 0.37	*p =* 0.36
Magnesium (mg)	206.3	14.9	276.9	28.9	200.0	9.6	190.3	17.4	*p =* 0.25	***p=*** **0.02**	***p=*** **0.04**
Manganese (mg)	1.8	0.4	1.4	0.7	1.8	0.2	1.2	0.4	*p =* 0.38	*p =* 0.99	*p =* 0.83
Potassium (mg)	2,344.2	160.5	3,011.8	312.0	2,198.4	104.0	2,242.1	187.2	*p =* 0.19	***p=*** **0.03**	*p =* 0.14
Selenium (μg)	34.3	6.4	28.3	12.5	36.7	4.2	32.2	7.5	*p =* 0.67	*p =* 0.56	*p =* 0.92
Sodium (mg)	2,532.2	272.4	2,429.1	529.4	2,823.7	176.5	2,602.9	317.6	*p =* 0.38	*p =* 0.50	*p =* 0.92
Zinc (mg)	12.6	1.7	22.1	3.3	12.0	1.1	13.0	2.0	*p =* 0.26	***p=*** **0.03**	*p =* 0.08

*More details of isolated effects of the adherence to dietary program and work shift in Supplementary Table 2. Bold values mean statistically significant*.

Table 4 also presents a significant effect of the interaction between shift and the adherence to nutritional counseling on the consumption of vitamin B12, C, and magnesium. The intake of these nutrients was higher in shift workers who adhered to dietary guidance in relation to shift workers who did not adhere (LSD *p* ≤ 0.01). The same was demonstrated in relation to day workers (LSD *p* ≤ 0.03). The consumption of vitamin B6 was also higher among evening and night workers who adhered to dietary guidance in relation to the morning workers who adhered (LSD *p* < 0.01) and who did not adhere (LSD *p* < 0.01) (Table 4).

**Figure 3:**
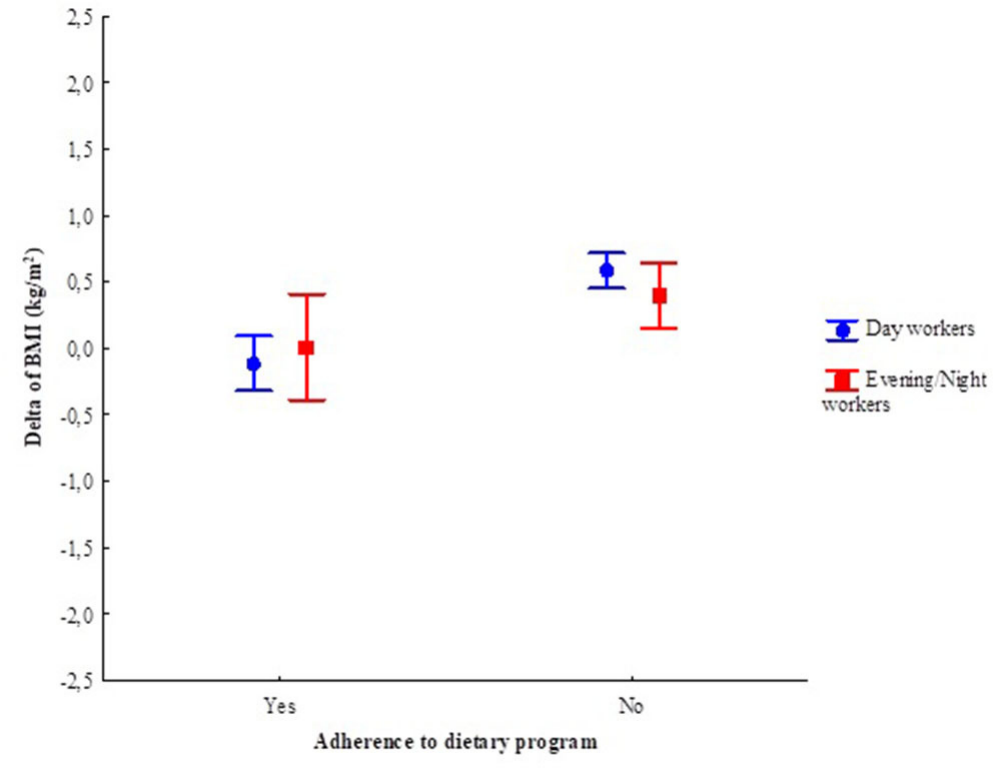
Generalized linear model of the BMI delta (pre- and during the pandemic period), according to the work shift and adherence to dietary program. Adjusted by age and sex. Work shift: *F*_(1, 142)_ = 0.06. *p* = 0.81; Adherence: *F*_(1, 142)_ = 16.79. *p* < 0.01; Adherence *Work shift: *F*_(1, 142)_ = 1.35. *p* = 0.25. Vertical bars indicate the 95% confidence interval. More details in Supplementary Table 3. *Adherence and interaction.

There was no interaction effect between shift and adherence to dietary program in the BMI delta (during the pandemic–pre-pandemic) (Figure 3). However, there was an isolated effect of adherence to dietary counseling, in which those who adhered had a BMI reduction of 0.07 kg/m^2^ (EP 0.11 kg/m^2^) compared with those who did not adhere had an increase of 0.49 kg/m^2^ (EP 0.07 kg/m^2^).

**Figure 4:**
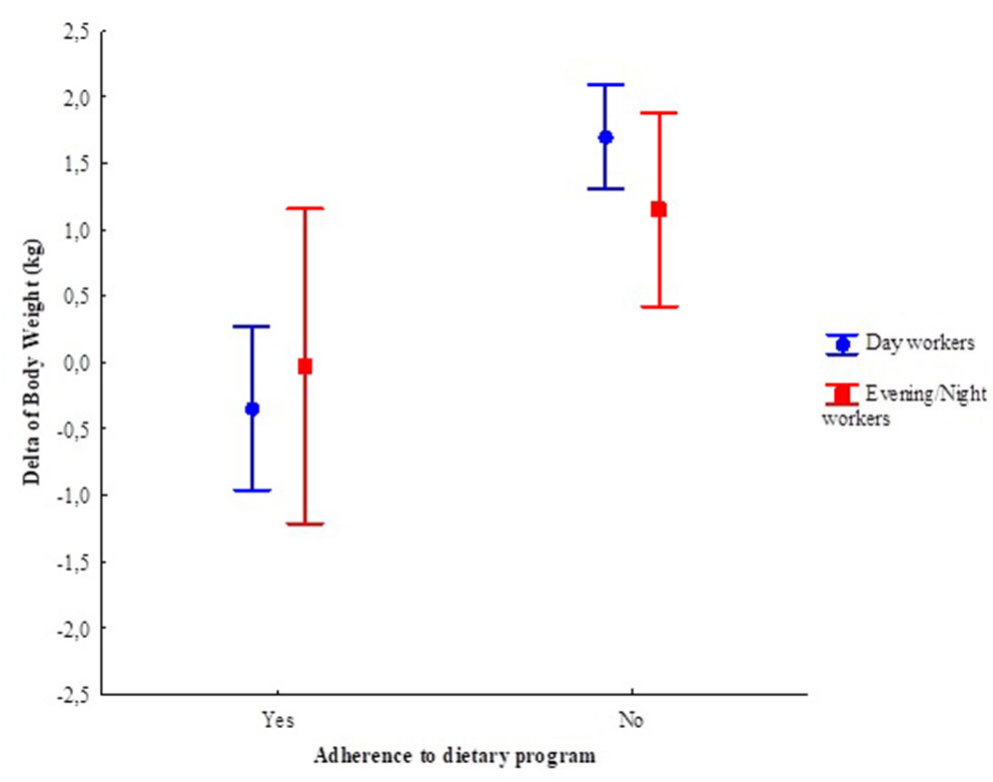
Generalized linear model (GLM) of the delta of body weight (pre- and during the pandemic), according to the work shift and adherence to dietary program. Adjusted by age and sex. Work shift: *F*_(1, 142)_ = 0.08. *p* = 0.78; Adherence: *F*_(1, 142)_ = 16.81, *p* < 0.01; Adherence *Work shift: *F*_(1, 142)_ = 1.22, *p* = 0.27. Vertical bars indicate the 95% confidence interval. More details in Supplementary Table 4. *Adherence and interaction.

There was also no interaction effect between shift and adherence to dietary program in the delta of body weight (pre-pandemic and during the pandemic) (Figure 4). However, there was an isolated effect of adherence to dietary counseling, in which those who adhered had a weight reduction of 0.21 kg (EP 0.33 kg), whereas those who did not adhere had an increase of 1.43 kg (EP 0.20 kg).

## Discussion

This study evaluated the impact of the COVID-19 pandemic on the dietary intake of day and shift workers and measured adherence to a nutritional counseling program and its effect on workers' food intake and body weight. As we hypothesized, the pandemic significantly increased the dietary intake of workers of both shifts. However, contrary to our initial hypothesis, we found that shift workers (evening and night) did not have less adherence to the nutritional counseling program compared with day workers. Furthermore, the nutritional counseling program impacted the dietary intake and body weight of workers on both shifts in the same way. Adherence to the nutritional counseling program had an impact on the consumption of proteins and some micronutrients, and also promoted a reduction in body weight and BMI of workers of both shifts. In addition, we found an isolated effect of the shift on the meal times, showing that evening/night workers ate their meals later than day workers. Interestingly, these shift workers anticipated the afternoon snack during the pandemic compared with the pre-pandemic, while day workers maintained their usual meal times. Taken together, these results emphasize the importance of working to improve workers' adherence in programs that emphasize behavioral change. We highlight the importance of a nutritional counseling program during such a critical period in terms of deteriorating lifestyle as the COVID-19 pandemic.

However, the cause of weight gain is complex and multifactorial, dietary habits, and lifestyle plays an important role in developing obesity (Chin and Chang, 2005; Danielsen et al., 2013), especially in shift workers, who are known to consume a poor diet (Antunes et al., 2010). To effectively treat nutritional disorders with diet management, controlling the excessive nutrient intake but also concretely instructing the know-how of choosing the right foods and desirable eating patterns are necessary (Danielsen et al., 2013). In addition, it is important to acquire the ability to control one's own dietary habits through continuous nutrition education and counseling (Kim et al., 2017). An important result of this study is that nutritional intervention at a time as difficult as the COVID-19 pandemic benefited a group of workers that were affected by nutritional problems (Alexander et al., 2020; Hill et al., 2021), such as shift workers (Crispim et al., 2011; Peplonska et al., 2019). The adherence to nutritional counseling did not differ between work shifts and proved to be effective in achieving the objectives of nutritional care regardless of work hours. This shows that the effectiveness of nutritional interventions in companies seems to be more focused on strategies on how to improve adherence than making it according to the shift. Although shift workers show specific eating patterns (Gusto et al., 2015) and health demands (Moreno et al., 2019), a good strategy for adhering to the dietary guidance may apply to everyone.

The results from this study showed that evening/night workers presented a different pattern of meal timing, having their meals later than day workers. Previous studies already showed that shift workers usually presenting a longer eating window (Marot et al., 2020), which indicates a high consumption of energy during the night shift (Shaw et al., 2019). Since food is considered a Zeitgeber, synchronizing the peripheral clocks, having meals at later phases of the internal biological clock may lead to a circadian misalignment (Wehrens et al., 2017). Interestingly, our results showed that the pandemic anticipated one of the meals of the day in shift workers—the afternoon snack—while day workers maintained their usual meal timings (see Supplementary Table 5). We believe that the pandemic moment may have given shift workers more free time to eat at times that meet physiological rather than environmental cues. These and other results of ours—such as the higher consumption of important nutrients (vitamin B12, C, B6, and magnesium) among workers of both shifts who adhered to the dietary program in relation to those who did not adhere—may have occurred due to individual nutritional counseling that focused on the habits and routine of the worker. These data reinforce the importance of nutritional guidance in the workplace in improving eating behavior in different aspects.

In addition to our proposal in this study, previous studies have analyzed the effect of workplaces' lifestyle interventions on the health promotion and dietary outcomes of workers (Ni Mhurchu et al., 2010; Schroer et al., 2014). In general, these interventions have been shown to improve employee health, increase productivity and be cost effective (U.S. Department of Health and Human Services, 2003) and many are tailored to suit the specific operational and organizational requirements of different workforces (Atlantis et al., 2006; Goetzel et al., 2010; Hutchinson and Wilson, 2012; Carpenter et al., 2014). Regarding workplace health promotion interventions for shift workers, Demou et al. conducted a systematic review to identify adaptations and intervention components that accommodate shift working and to assess their impact on weight, physical activity, sedentary behavior, and healthy eating. Authors demonstrated that group-based workplace interventions can be effective for supporting shift workers to lose weight and increase physical activity. However, the authors pointed out that it is also important that group-based workplace interventions to promote changes in the lifestyle of shift workers consider a number of adaptations at the organizational level, including flexible delivery, the proximity of intervention sites to the workplace, and management support and encouragement (Demou et al., 2018).

This study has limitations. Subjective dietary evaluation depends on the memory and motivation of the participants, which is susceptible to errors. To minimize this bias, a trained nutritionist conducted all interviews, in order to minimize the omission of possibly forgotten foods and standardize the level of detail for describing foods. Moreover, the interviews were applied in a silent room only with the presence of the interviewer and the worker to minimize the influence of psychological determinants. An atlas of illustrations was used to specify the information to the workers. Additionally, all the inconsistencies were checked and confirmed (e.g., content and timing of food intake). This study utilized a convenience sample of workers, which may limit the results. However, day workers were a control group to reduce such limitations. Another limitation is the imbalance between the number of people in the groups of workers on day shifts, which is very common in the literature because the vast majority of companies and services employ more day workers than shift workers. Finally, data collection took place mainly in the summer in the pre-pandemic period and winter in the post-pandemic period. However, despite the known relationship between seasonality and food consumption (Stelmach-Mardas et al., 2016), we believe that this factor has little influence on our results, as the winter in the city where the study was conducted was very mild in 2020. On the other hand, this study has strengths as the proposal for an intervention in the workplace and the design followed workers over a period as important as this, in this pandemic.

In conclusion, the pandemic seems to contribute to the increase in food intake of workers, regardless of the work shift. Those who joined a nutritional counseling program managed their food intake and lost weight. These changes in the dietary pattern resulting from adherence to nutritional counseling can be decisive to treat and prevent the increase in obesity during the pandemic. More studies with larger samples should confirm these results.

## Data Availability Statement

The original contributions presented in the study are included in the article/Supplementary Material, further inquiries can be directed to the corresponding author/s.

## Ethics Statement

The studies involving human participants were reviewed and approved by Research Ethics Committee of the Faculty of Public Health of the University of São Paulo (Process# 4298715). The patients/participants provided their written informed consent to participate in this study.

## Author Contributions

PN contributed to concept/design, data collection, original draft, and data curation and edited and reviewed the manuscript. LM contributed to data curation, original draft, analysis, and interpretation of data and edited and reviewed the manuscript. LN contributed to data curation and original draft and edited and reviewed the manuscript. EM, CC, and CM contributed to concept/design, project administration, original draft, analysis, and interpretation of data and edited and reviewed the manuscript. All authors have read and approved the manuscript.

## Conflict of Interest

The authors declare that the research was conducted in the absence of any commercial or financial relationships that could be construed as a potential conflict of interest.
