# Supplementary material for: A Nutritional Counseling Program Prevents an Increase in Workers' Dietary Intake and Body Weight During the COVID-19 Pandemic
Source: Front Physiol. 2021 Jul 21;12:703862. doi: 10.3389/fphys.2021.703862 (PMC8335487; doi:10.3389/fphys.2021.703862)
Supplement: Supplementary file 1 [file Table_1.PDF]

Supplement 1 – Statistically significant isolated effects of the pandemic and work shift on the nutrient consumption, adjusted for age and sex (Generalized linear model).

**Nutrients**	**Pre-pandemic**		**Pandemic**		**Day**		**Evening/night**	
	**mean**	**SE**	**mean**	**SE**	**mean**	**SE**	**mean**	**SE**
Energy (kcal)	1,334.02	59.95	1,843.19	59.95				
Protein (g)	78.32	4.58	109.60	4.58				
Carbohydrate (g)	152.59	7.39	200.71	7.39				
Total fiber (g)	12.90	0.74	15.61	0.74				
Total fat (g)	46.39	2.81	64.18	2.81				
Saturated fat (g)	19.29	1.37	26.07	1.37				
Monounsaturated (g)	16.01	1.07	20.94	1.07				
Polyunsaturated fat (g)	9.70	0.77	12.38	0.77				
Cholesterol (mg)	323.44	28.34	448.33	28.34				
Vitamin A (UI)	336.49	109.77	648.07	109.77				
Vitamin B2 (mg)	0.74	0.05	1.03	0.05				
Pantothenic acid (mg)	1.01	0.08	1.37	0.08				
Vitamin B6 (mg)	0.63	0.06	1.02	0.06				
Vitamin B12 (mg)	1.41	0.19	2.29	0.36				
Vitamin C (mg)	59.31	14.13	166.16	25.99				
Vitamin E (mg)	3.57	0.55	5.10	0.55	5.03	0.37	3.63	0.69
Niacin (mg)	14.37	1.44	22.35	1.44				
Copper (mg)	0.75	0.08	0.99	0.08				
Folate (µg)	54.38	5.13	76.99	5.13	52.76	3.46	78.61	6.37
Iron (mg)	64.73	16.28	19.35	16.28				
Phosphor (mg)	922.98	53.42	1,236.47	53.42				
Magnesium (mg)	161.94	7.33	207.55	7.33				
Potassium (mg)	1,779.22	81.40	2,343.66	81.40				
Sodium (mg)	1,988.45	127.66	2,647.23	127.66				
Zinc (mg)	9.90	0.84	13.78	0.84				
