# Supplementary material for: A Nutritional Counseling Program Prevents an Increase in Workers' Dietary Intake and Body Weight During the COVID-19 Pandemic
Source: Front Physiol. 2021 Jul 21;12:703862. doi: 10.3389/fphys.2021.703862 (PMC8335487; doi:10.3389/fphys.2021.703862)
Supplement: Supplementary file 2 [file Table_2.PDF]

Supplement 2 – Statistically significant isolated effects of the adherence to dietary program and work shift on the nutrient consumption, adjusted for age and sex (Generalized linear model).

Nutrients	Adhered the program		Did not adhere the program		Day		Evening/night	
	Mean	SE	Mean	SE	Mean	SE	Mean	SE
Protein (g)	126.0	9.8	103.5	6.0				
Vitamin B6 (mg)	1.4	0.1	0.9	0.1	0.9	0.1	1.4	0.1
Vitamin B12 (mg)	3.9	0.6	1.6	0.3	1.7	0.3	3.9	0.6
Vitamin C (mg)	220.9	40.2	99.5	24.6	66.1	21.9	254.3	41.7
Folate (µg)					60.2	6.1	101.9	11.5
Magnesium (mg)	241.6	16.3	195.2	9.9				
Potassium (mg)	2,678.0	175.5	2,220.2	107.1				
Zinc (mg)	17.3	1.9	12.5	1.1				
