# Supplementary material for: A Nutritional Counseling Program Prevents an Increase in Workers' Dietary Intake and Body Weight During the COVID-19 Pandemic
Source: Front Physiol. 2021 Jul 21;12:703862. doi: 10.3389/fphys.2021.703862 (PMC8335487; doi:10.3389/fphys.2021.703862)
Supplement: Supplementary file 3 [file Table_3.PDF]

Supplement 3 –BMI delta (pre-pandemic period and during the pandemic) by work shift, adherence to dietary program and interaction of shift and adherence, adjusted for age and sex (Generalized linear model).

**Variables**		**Mean**	**SE**	**CI -95%**	**CI +95%**	**n**
**Shifts**						
	Day	0.24	0.06	0.12	0.36	114
	Evening/night	0.18	0.12	-0.05	0.41	34
**Adherence to the program**						
	Yes	-0.07	0.11	-0.29	0.15	43
	No	0.49	0.07	0.35	0.62	105
**Shift**	**Adherence**					
Day	*Yes*	-0.11	0.10	-0.32	0.09	34
Day	*No*	0.59	0.07	0.46	0.72	80
Evening/night	*Yes*	-0.02	0.20	-0.42	0.37	9
Evening/night	*No*	0.39	0.12	0.15	0.62	25
