# Supplementary material for: A Nutritional Counseling Program Prevents an Increase in Workers' Dietary Intake and Body Weight During the COVID-19 Pandemic
Source: Front Physiol. 2021 Jul 21;12:703862. doi: 10.3389/fphys.2021.703862 (PMC8335487; doi:10.3389/fphys.2021.703862)
Supplement: Supplementary file 4 [file Table_4.PDF]

Supplement 4 – Delta of body weight (pre-pandemic period and during the pandemic) by the work shift, adherence to dietary program and interaction of the shift and adherence, adjusted for age and sex (Generalized linear model).

**Variables**		**Mean**	**SE**	**CI -95%**	**CI +95%**	**n**
**Shifts**						
	Day	0.67	0.18	0.31	1.03	114
	Evening/night	0.54	0.35	-0.14	1.23	34
**Adherence to the program**						
	Yes	-0.21	0.33	-0.87	0.44	43
	No	1.43	0.20	1.03	1.83	105
**Shift**	**Adherence**					
Day	*Yes*	-0.36	0.30	-0.96	0.24	34
Day	*No*	1.71	0.20	1.31	2.10	80
Evening/night	*Yes*	-0.07	0.59	-1.24	1.10	9
Evening/night	*No*	1.16	0.36	0.45	1.86	25
