# Supplementary material for: A Nutritional Counseling Program Prevents an Increase in Workers' Dietary Intake and Body Weight During the COVID-19 Pandemic
Source: Front Physiol. 2021 Jul 21;12:703862. doi: 10.3389/fphys.2021.703862 (PMC8335487; doi:10.3389/fphys.2021.703862)
Supplement: Supplementary file 5 [file Table_5.PDF]

Supplement 5 – Generalized linear model of the variables related to the meal timing in the pre-pandemic period and during the pandemic per work shift, adjusted for age and sex.

Meal timing	Pre-pandemic				Pandemic				Shift Effect	Pandemic Period Effect	Interaction Shift*Pandemic
	Day shift		Evening/night shift		Day shift		Evening/night shift				
	**Mean**	**SE**	**Mean**	**SE**	**Mean**	**SE**	**Mean**	**SE**			
Breakfast	07:22	00:05	09:07	00:10	07:26	00:05	09:14	00:10	**p<0.01**	p=0.48	p=0.87
Morning snack	09:52	00:07	14:37	00:58	09:40	00:07	13:37	00:42	**p<0.01**	p=0.33	p=0.51
Lunch	12:18	00:04	14:06	00:09	12:13	00:05	13:55	00:09	**p<0.01**	p=0.26	p=0.64
Afternoon snack	15:54	00:11	18:06	00:32	16:07	00:10	16:46	00:27	**p<0.01**	p=0.12	**p=0.03**
Dinner	20:10	00:08	20:48	00:15	19:50	00:08	20:42	00:15	**p<0.01**	p=0.27	p=0.57
Night snack	21:52	00:27	01:19	00:22	22:02	00:16	00:25	00:25	**p<0.01**	p=0.33	p=0.16
